# Application of Plasticizer Glycerol in Lignosulfonate-Filled Rubber Compounds Based on SBR and NBR

**DOI:** 10.3390/ma16020635

**Published:** 2023-01-09

**Authors:** Ján Kruželák, Klaudia Hložeková, Andrea Kvasničáková, Michaela Džuganová, Ivan Chodák, Ivan Hudec

**Affiliations:** 1 Department of Plastics, Rubber and Fibres, Faculty of Chemical and Food Technology, Slovak University of Technology in Bratislava, Radlinského 9, 812 37 Bratislava, Slovakia; 2 Polymer Institute, Slovak Academy of Sciences, Dúbravská Cesta 9, 845 41 Bratislava, Slovakia

**Keywords:** rubber compounds, calcium lignosulfonate, glycerol, cross-linking, physical–mechanical properties, morphology

## Abstract

The work deals with the application of biopolymer fillers in rubber formulations. Calcium lignosulfonate was incorporated into styrene–butadiene rubber and acrylonitrile–butadiene rubber in a constant amount of 30 phr. Glycerol in a concentration scale ranging from 5 to 20 phr was used as a plasticizer for rubber formulations. For the cross-linking of the compounds, a sulfur-based curing system was used. The study was focused on the investigation of glycerol in the curing process; the viscosity of rubber compounds; and the cross-link density, morphology, physical–mechanical, and dynamic mechanical properties of vulcanizates. The study revealed that the application of glycerol as a plasticizer resulted in a reduction in the rubber compounds’ viscosity and contributed to the better dispersion and distribution of the filler within the rubber matrices. The mutual adhesion and compatibility between the filler and the rubber matrices were improved, which resulted in the significant enhancement of tensile characteristics. The main output of the work is the knowledge that the improvement of the physical–mechanical properties of biopolymer-filled vulcanizates can be easily obtained via the simple addition of a very cheap and environmentally friendly plasticizer into rubber compounds during their processing without additional treatments or procedures. The enhancement of the physical–mechanical properties of rubber compounds filled with biopolymers might contribute to the broadening of their potential applications. Moreover, the price of the final rubber articles could be reduced, and more pronounced ecological aspects could also be emphasized.

## 1. Introduction

Lignin is a natural polymer and one of the three basic elements of wood, together with cellulose and hemicellulose. It is the second most widespread biological material in the word after cellulose. Lignin is a complex three-dimensional aromatic polymer that most often occurs in coniferous and leafy wood but can also be found in annual plants [1,2]. Lignin molecules are formed from atoms of carbon, hydrogen, and oxygen. The elementary composition of lignin is not constant; rather, it considerably depends on its source and isolation method. The average amount of carbon is around 60%, the average amount of hydrogen is 6%, and the average amount of oxygen is 30%. Paracoumaryl alcohol, sinapyl alcohol, and coniferyl alcohol are lignin precursors from which phenyl propane units are formed, namely p-hydroxyphenyl (H), syringyl (S), and guaiacyl (G) units, respectively. The radical copolymerization of these units leads to the formation of lignin polymers [3,4,5]. Regarding structure, lignin is an amorphous, highly branched polymer containing ample amounts of functional groups, mostly methoxyl, phenolic, and aliphatic hydroxyl moieties. The side chains mainly comprise carbonyl or carboxylic groups [6,7]. These active sites are very important for chemical and biological reactions and affect the reactivity of lignin. Lignin is a by-product of the paper and pulp industry. To utilize lignin, it needs to be isolated from a biomass by using various processes [8]. Several extraction and delignification processes with acid- or alkali-catalyzed mechanisms are used to obtain so-called technical lignins [9,10,11,12]. Technical lignins can be divided into two groups. The first group comprises lignins containing sulfur such as Kraft lignins and lignosulfonates, which are produced in high amounts. The second group are sulfur-free lignins, such as alkalignins, which are derived from various fractionating processes. The Kraft pulping and sulfite extraction processes are currently the most frequently used processes for lignin production. Kraft pulping is performed in an aqueous alkali medium using sodium hydroxide and sodium sulfide [9,13]. The sulfite pulping is carried out in an acidic environment using aqueous sulfur dioxide and a suitable salt-based acid [11,14]. Sulfite lignins, known as lignosulfonates, are considerably different from Kraft lignins in terms of their composition and properties. They contain higher amounts of sulfur and smaller amounts of phenolic and carboxylic groups. They are also more widely utilized, as the sulfite process imparts lignosulfonate with ion exchange, emulsifying, deflocculation and dispersive properties [15,16].

Nowadays, there is an increasing effort to utilize lignins. Still, only around 2% of the overall annually produced lignin is commercially used. The rest is usually incinerated to obtain energy or just landfilled. Lignins have many interesting properties, such as biodegradability, non-toxicity, antimicrobial and antioxidant behavior, high availability, very good adsorption, and adhesion [17,18,19]. They are also compatible with several chemical compounds because they contain many reactive sites that enable various substitutive and addition reactions. Their aromatic structure ensures good stability and mechanical properties. Those properties make lignins suitable for a wide sphere of applications, such as adhesives, flocculants, surfactants, dispersant agents, compatibilizers, stabilizers, flame retardants, binders, additives to composites, in energy storage, and 3D printing applications [8,20,21,22,23,24,25]. Their high amount of carbon, mechanical stability, and good rheological and viscoelastic properties also make them suitable candidates as additives and fillers for rubber compounds [1,26,27,28,29,30,31,32]. The biggest problem arising from the application of lignins in rubber formulations is the deterioration of the physical–mechanical properties of the final products, mostly due to the poor homogeneity and compatibility between the rubber and the filler in the rubber–filler interface. Various chemical and/or physical techniques and modification treatments have been developed to improve the mutual compatibility between both components [33,34,35,36,37]. Following these procedures, materials with a high added value can be prepared, thus clearly demonstrating the high application potential of lignins in rubber compounds. However, many of these techniques require additional expenses and/or are time consuming. 

Styrene–butadiene rubber (SBR) is a general-purpose rubber, and it is the most widely used material in rubber technology, mostly for the production of tires, conveyor belts, hoses, sealings, shoe soles, and rubber flooring. It is a non-polar, unsaturated rubber consisting of butadiene and styrene structural units. Increasing the amount of styrene in rubber chains leads to increases in tensile and tear strength, improved processability, and ageing resistance. On the other hand, elasticity is reduced and the glass transition temperature increases. SBR has generally weak tensile characteristics, and active fillers must be used to improve the mechanical properties of the final products. 

Acrylonitrile–butadiene rubber (NBR) is specialty type rubber very frequently used in many technical applications, such as adhesives, membranes, sealings, rubber flooring, hoses, cable sheeting, conveyor belts, and many small parts in automotive industry. It is a copolymer of butadiene and acrylonitrile. As acrylonitrile is a polar structural unit, NBR is a polar rubber. Its special feature is oil resistance, which increases with increasing amounts of acrylonitrile. When unfilled with reinforcing fillers, it also exhibits low mechanical properties such as low tensile and tear strength.

Glycerol is a colorless, odorless, hydrophilic, and viscous liquid. It has antimicrobial and antiviral properties. Glycerol is easily soluble in water due to the ability of its polyol groups to form hydrogen bonds with water molecules. Glycerol is used in a number of industrial applications; in the pharmaceutical industry; in cosmetics and personal care products; as a humectant in food; in the production of resins, detergents, plastics, and tobacco; and as a plasticizer. 

In this work, calcium lignosulfonate was incorporated into compounds based on SBR and NBR. Glycerol was used as a cheap and eco-friendly plasticizer to improve the dispersion of lignosulfonate within the rubber matrices and to improve the adhesion and homogeneity between the rubber and the filler in the filler–rubber interface. 

## 2. Experimental

### 2.1. Materials

Styrene–butadiene rubber (SBR) of the Kralex 1502 type (styrene content of 23.5%) produced via cold emulsion polymerization was provided by Synthos Kralupy, Kralupy nad Vltavou, Czech Republic. Acrylonitrile–butadiene rubber (NBR) of the SKN 3345 type (acrylonitrile content of 31–35%) was supplied by Sibur International, Moscow, Russia. Calcium lignosulfonate, with the trade name Borrement CA120, was supplied by Borregaard Deutschland GmbH, Karlsruhe, Germany. The specific surface area of the filler was 3.9 m^2^·g^−1^, and its average molecular weight was 24,000 g·mol^−1^. An elemental analysis revealed the presence of carbon (46.63 wt.%), nitrogen (0.14 wt.%), hydrogen (5.35 wt.%), sulfur (5.62 wt.%) and hydroxyl groups (1.56 wt.%) in its structure. The biopolymer filler was incorporated into rubber compounds at a constant amount of 30 phr. Glycerol (86% solution) as a plasticizer was supplied by Sigma-Aldrich, St. Louis, MO, USA. Glycerol was applied to the rubber formulations in a concentration scale ranging from 5 to 20 phr. For the cross-linking of the rubber compounds, a sulfur-based curing system consisting of zinc oxide and stearic acid (Slovlak, Košeca, Slovakia) as activators, N-cyclohexyl-2-benzothiazole sulfenamide (CBS) (Duslo, Šaľa, Slovakia) as an accelerator, and sulfur (Siarkopol, Tarnobrzeg, Poland) was used. 

### 2.2. Methods

#### 2.2.1. Preparation and Curing of Rubber Compounds

Styrene–butadiene rubber and acrylonitrile–butadiene rubber were filled with calcium lignosulfonate, which was kept constant in all rubber formulations at 30 phr. Glycerol was applied to rubber formulations in a concentration scale ranging from 5 to 20 phr. A sulfur-based curing system was used for the curing and cross-linking of the rubber compounds. 

The fabrication of the rubber compounds proceeded in a two-step mixing process in a laboratory kneading machine Brabender (Brabender GmbH & Co. KG, Duisburg, Germany). The compounding temperature was set to 90 °C with a rotor speed of 55 rpm. The overall mixing process took 10 min. First, the rubber was plasticated for 1 min, and then zinc oxide and stearic acid were added for the next 1 min of compounding. Subsequently, the filler was incorporated, glycerol was introduced after 2 min, and the mixing process continued for another 2 min. The rubber compounds were taken out from the mixing chamber and cooled down in a two-roll mill. In the second step, which took 4 min at 90 °C and 55 rpm, sulfur and the CBS accelerator were applied. In the final stage, the rubber compounds were homogenized and sheeted in the two-roll mill. 

The curing process of the rubber compounds was performed at a temperature of 170 °C and a pressure of 15 MPa in a Fontijne hydraulic press (Fontijne, Vlaardingen, The Netherlands) according to their optimum cure times. After curing, thin sheets with dimensions of 15 × 15 cm and a thickness of 2 mm were obtained.

#### 2.2.2. Determination of Curing Characteristics

The curing characteristics of the rubber compounds were determined from corresponding curing isotherms, which were investigated with an MDR 2000 oscillatory rheometer (Alpha Technologies, Akron, OH, USA).

The investigated curing parameters were:

M_L_—minimum torque (dN·m).
M_H_—maximum torque (dN·m).
∆M—torque difference, the difference between M_H_ and M_L_ (dN·m).
t_c90_—optimum curing time (min).
t_s1_—scorch time (min).

#### 2.2.3. Determination of Cross-Link Density 

The cross-link density (*ν*) was determined based on the equilibrium swelling of the vulcanizates in xylene. The weighted dried samples were placed into xylene, in which they swelled over time. The weight of samples was measured every hour until equilibrium swelling was reached. During the measurement, the solvent diffused into the rubber and disrupted almost all physical interactions in the rubber matrix. The result was the determination of the chemical cross-link density, i.e., the concentration of the chemical cross-links within the rubber compounds. The experiments were carried out at a laboratory temperature, and the swelling time was equal to 30 h. The Flory–Rehner equation modified by Krause [38] was then used to calculate the cross-link density based on the equilibrium swelling state. 

#### 2.2.4. Rheological Measurements

The dynamic viscosity of the rubber compounds was investigated using an RPA 2000 (Alpha Technologies, Akron, OH, USA). The samples were analyzed under strain amplitude from 0.15 to 700% at a constant frequency of 0.2 Hz and a temperature of 90 °C.

#### 2.2.5. Investigation of Physical–Mechanical Characteristics

A Zwick Roell/Z 2.5 appliance (Zwick GmbH & Co. KG, Ulm, Germany) was used to evaluate the tensile properties of the vulcanizates. The tests were performed in accordance with valid technical standards, and the cross-head speed of the measuring device was set to 500 mm·min^−1^. Dumbbell-shaped test samples (width of 6.4 mm, length of 80 mm, and thickness of 2 mm) were used for measurements. Hardness was measured by using a durometer and is expressed in the Shore A scale.

#### 2.2.6. Microscopic Analysis

The surface morphology and microstructure of the vulcanizates were observed using a JEOL JSM-7500F scanning electron microscope (Jeol Ltd., Tokyo, Japan). The samples were first cooled down in liquid nitrogen under their glass transition temperature and then fractured into small fragments with a surface area of 3 × 2 mm. The fractured surface was covered with a thin layer of gold and placed into the microscope. The electron source was a cold cathode UHV field emission gun, the acceleration voltage ranged from 0.1 kV to 30 kV, and the resolution was 1.0 nm at 15 kV and 1.4 nm at 1 kV. SEM images were captured with a CCD EDS Camera (Oxford INCA X-ACT). 

#### 2.2.7. Determination of Dynamic Mechanical Properties

The dynamic mechanical performance of the vulcanizates was calculated by using a MkIII DMTA dynamic mechanical analyzer from Rheometric Scientific (New Castle, DE, USA). The samples were analyzed in the tensile mode at a frequency 10 Hz, an amplitude of dynamic deformation of 64 μm, and a static force of 0.2 N in a temperature range from −60 °C to 80 °C. 

## 3. Results and Discussion

### 3.1. Curing Process and Cross-Link Density

**Figure 1:**
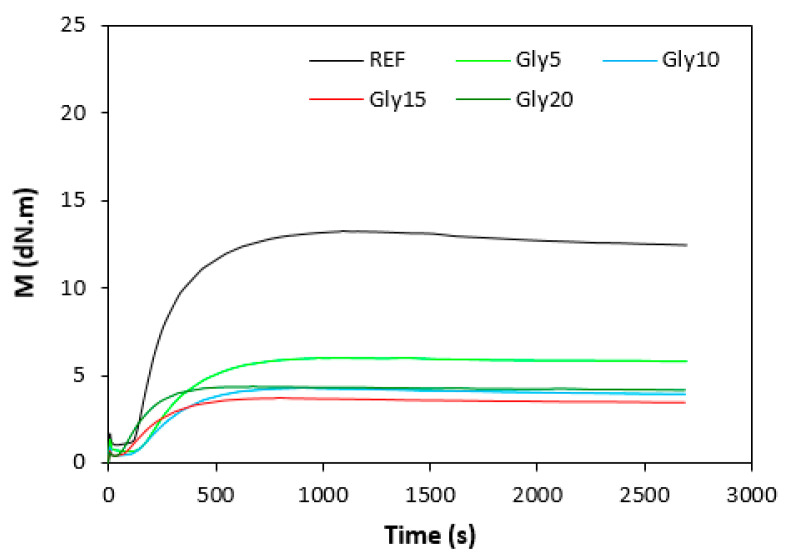
Vulcanization isotherms for rubber compounds based on SBR.

**Figure 2:**
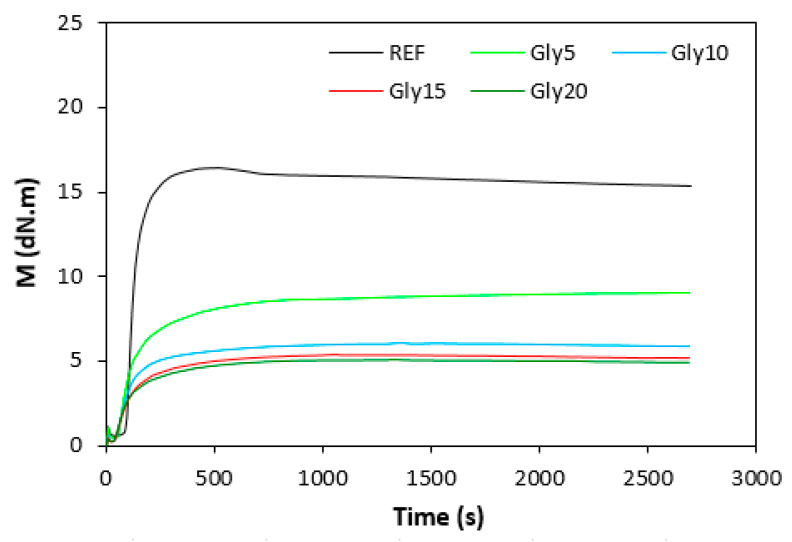
Vulcanization isotherms for rubber compounds based on NBR.

**Figure 3:**
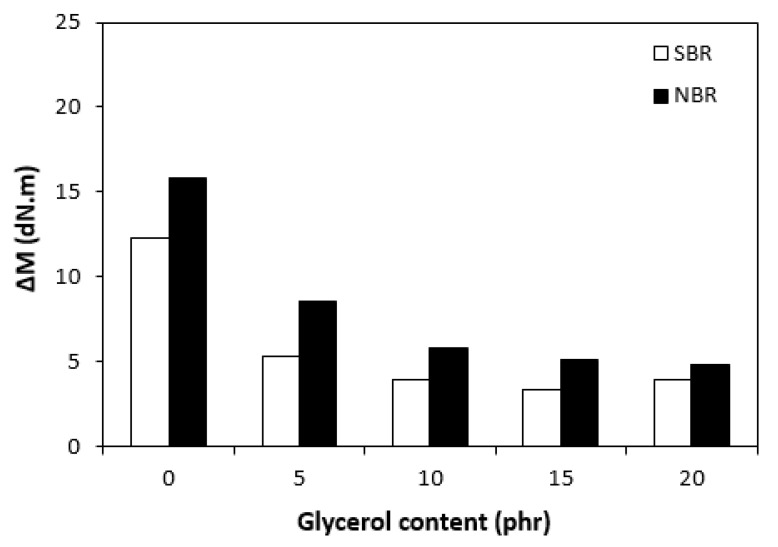
Influence of glycerol content on torque difference (ΔM) of rubber compounds.

**Figure 4:**
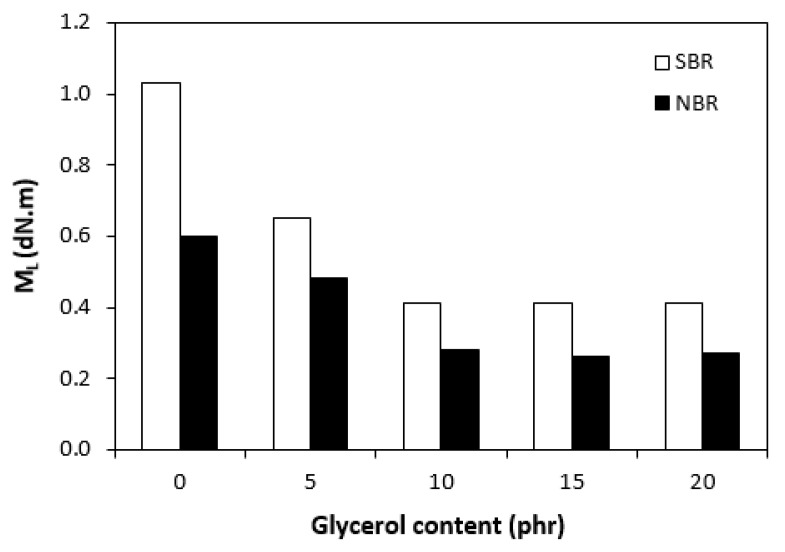
Influence of glycerol content on minimum torque (M_L_) of rubber compounds.

**Figure 5:**
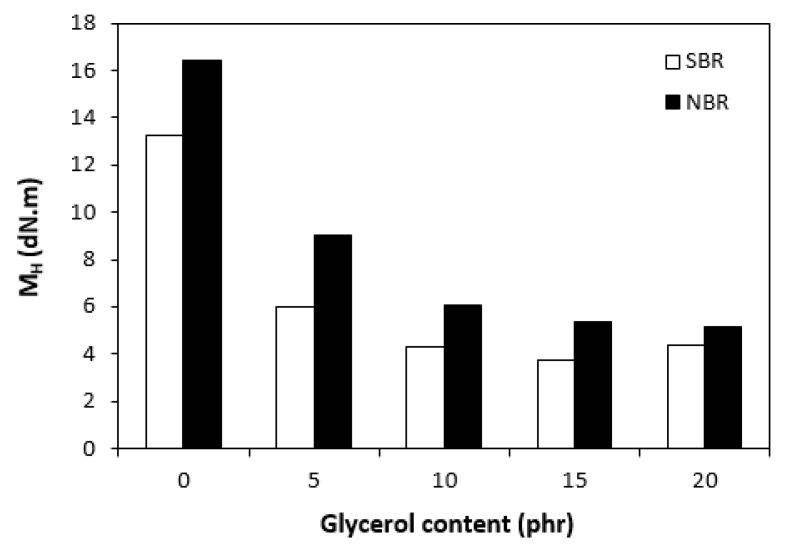
Influence of glycerol content on maximum torque (M_H_) of rubber compounds.

**Figure 6:**
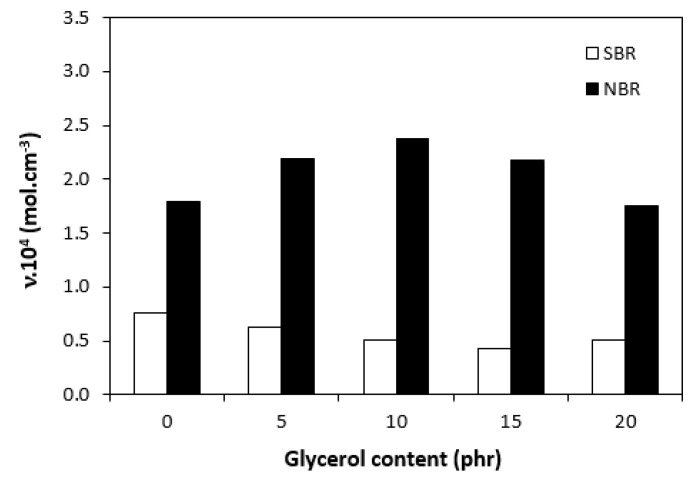
Influence of glycerol content on cross-link density (υ) of vulcanizates.

**Figure 7:**
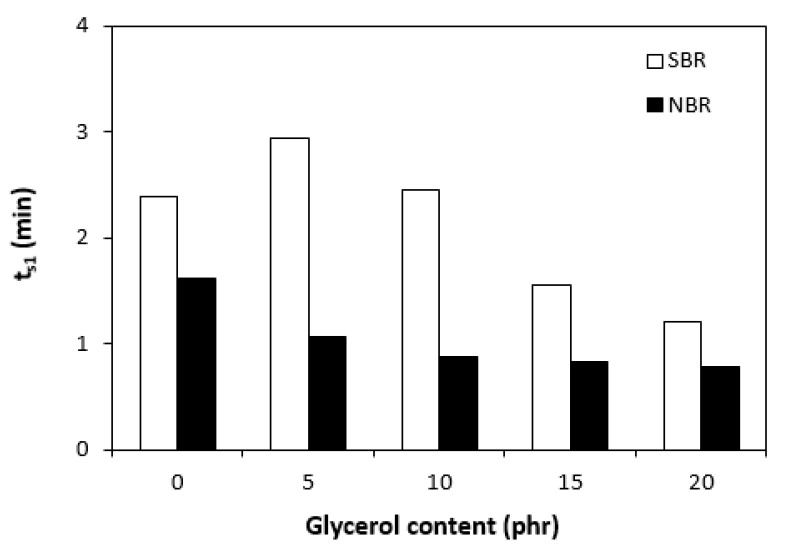
Influence of glycerol content on scorch time (t_s1_) of rubber compounds.

**Figure 8:**
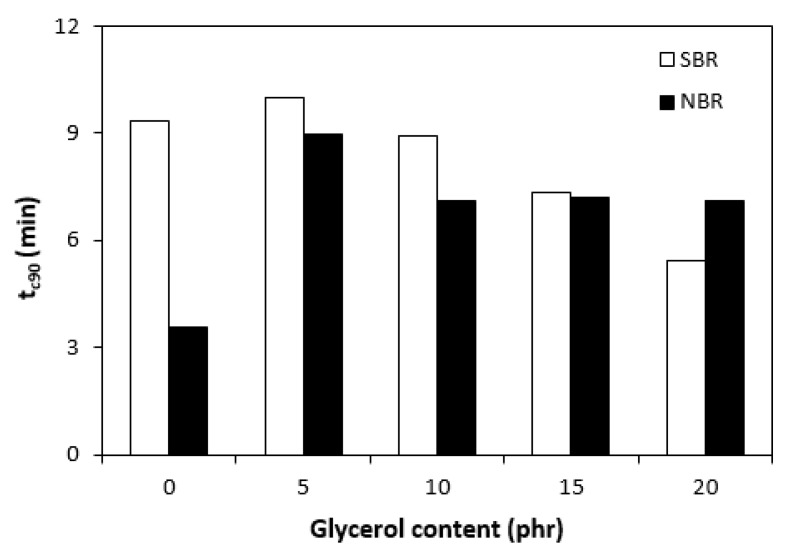
Influence of glycerol content on optimum cure time (t_c90_) of rubber compounds.

The curing isotherms for both types of rubber compounds obtained with the oscillatory rheometer are depicted in Figure 1 and Figure 2, which show that the presence of a plasticizer influenced the rate and state of the cure. The biggest differences between the maximum and minimum torque ΔM (ΔM = M_H_ − M_L_) were found in reference rubber compounds without glycerol (Figure 3). As the content of glycerol increased, the torque difference became smaller. The maximum M_H_ and minimum M_L_ torque for both rubber compound types were also found to decrease with increasing glycerol content (Figure 4 and Figure 5). The decrease in the maximum and minimum torque, as well as the torque difference, clearly suggested that glycerol had a strong plasticizing effect on the rubber compounds. Molecules of glycerol entered intermolecular space and disrupted the intra- and intermolecular physical forces and entanglements between rubber chains. This led to an increase in rubber chain mobility, a reduction in internal friction, and a lowering of the rubber compounds’ viscosity. It is interesting that though a higher minimum torque was found in the rubber compounds based on SBR, a higher maximum torque was found in the NBR-based rubber compounds. Minimum torque is usually related to the viscosity of rubber compounds before the curing process starts, which suggests that a higher viscosity was exhibited by the rubber compounds based on SBR. The results of the rheological measurements confirmed this presumption (see next chapter). On the other hand, maximum torque was found to be connected with the viscosity of the cured rubber compounds, which was also closely related with cross-link density. Higher maximum torque (M_H_) and higher torque difference (ΔM) values were found in the rubber compounds based on NBR at all studied glycerol concentrations, which indicates the higher cross-link density of the vulcanizates based on NBR. The results obtained from the experimental determination of cross-link density confirmed the presumption that the vulcanizates based on NBR exhibited a higher cross-link density (Figure 6). The cross-link density of the vulcanizates based on SBR slightly decreased with increase in glycerol content, while the cross-link density of NBR-based vulcanizates reached a slight maximum at 10 phr of glycerol and then dropped down. The application of glycerol led to a slight decrease in the scorch time (t_s1_) of the rubber compounds based on NBR (Figure 7). Similarly, the scorch time of the rubber compounds based on SBR (with the exception of rubber compound with 5 phr of a plasticizer) decreased with increases in the glycerol content. A very similar dependence was recorded for the optimum cure time (t_c90_) of the SBR-based rubber compounds. Following the initial rise of t_c90_ at 5 phr of glycerol, the rubber compounds with higher level of glycerol loading required less time for their optimum cross-linking compared with the reference (Figure 8). On the other hand, the lowest t_c90_ was found in the reference rubber compound based on NBR. The application of glycerol resulted in the prolongation of the optimum cure time for the NBR-based rubber compounds. These changes in the curing kinetics and prolongation of the optimum cure time can be attributed to changes in the shape and course of the curing isotherms due to the presence of glycerol. Another possible explanation is based on the assumption that glycerol, as a strong polar plasticizer, can dilute or absorb curing reagents and make them ineffective during the curing process. The lower the amount of curing additives, the lower the curing speed. However, this does not explain the decrease in t_c90_ for the rubber compounds based on SBR with a high glycerol content. Thus, further experiments are necessary to fully understand the influence of glycerol on the curing process of rubber compounds filled with lignosulfonate. 

### 3.2. Rheological Measurement

**Figure 9:**
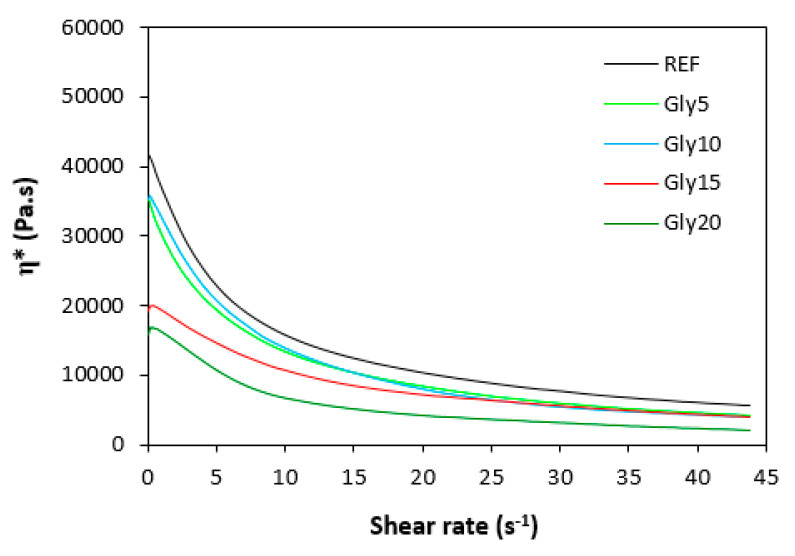
Dependence of dynamic complex viscosity (η*) of rubber compounds based on SBR on shear rate.

**Figure 10:**
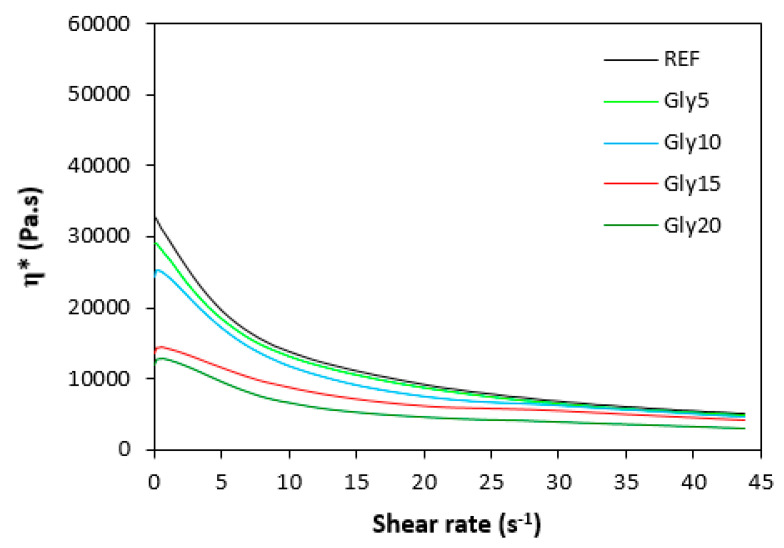
Dependence of dynamic complex viscosity (η*) of rubber compounds based on NBR on shear rate.

The dependences of the dynamic complex viscosity (η*) on the shear rate of the rubber compounds based on SBR are illustrated in Figure 9, and those for the rubber compounds based on NBR are presented in Figure 10. It is apparent that in both cases, the highest complex viscosity in the whole range of the shear rate was in the reference rubber compounds without glycerol. The higher the amount of glycerol, the lower the complex viscosity. The rubber compounds with the highest amount of plasticizers exhibited the lowest viscosity. The differences in viscosities were more pronounced at lower shear rates. With increases in shear rate, the differences in the viscosity dependence on glycerol content became smaller. Comparing both types of rubber compounds clearly showed that higher complex viscosities were found in the rubber formulations based on SBR. The rheological measurements were in good agreement with the experimental data summarized in previous section, clearly confirming that the application of glycerol resulted in a plasticizing effect on the rubber compounds and the lowering of their viscosities. The higher viscosities of the rubber compounds based on SBR were responsible for the higher minimum torque (M_L_) of the equivalent rubber compounds, while the higher cross-link densities of the vulcanizates based on NBR contributed to the higher maximum torque (M_H_) and, subsequently, the higher torque increment (ΔM) for the NBR-based rubber formulations. 

### 3.3. Physical–Mechanical Properties

**Figure 11:**
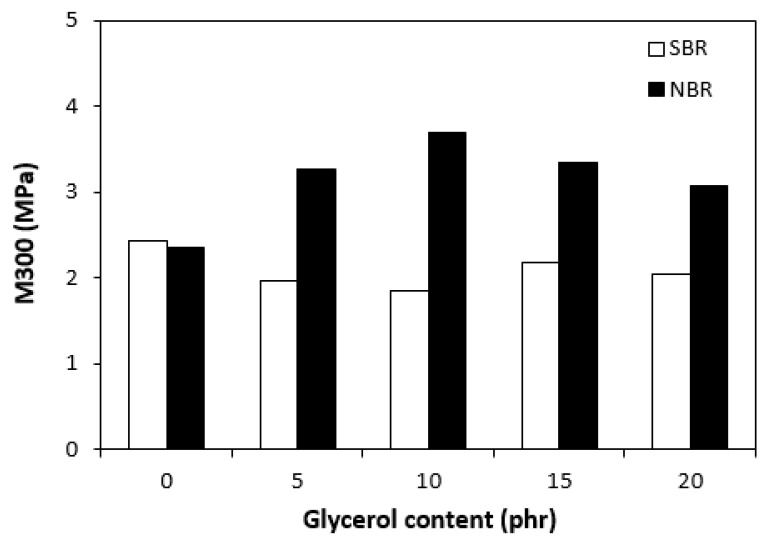
Influence of glycerol content on modulus (M300) of vulcanizates.

**Figure 12:**
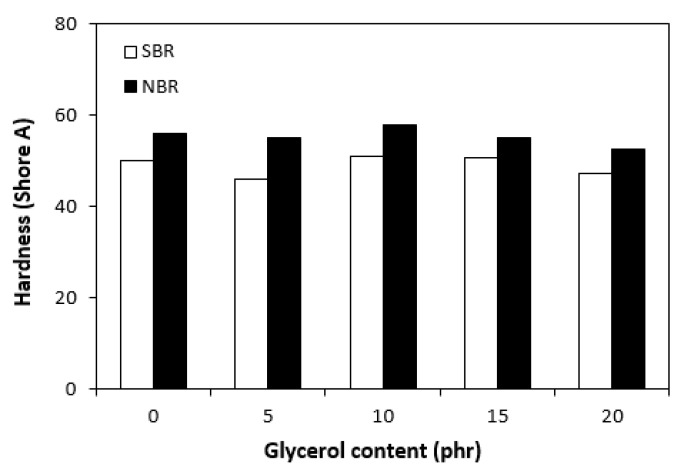
Influence of glycerol content on hardness of vulcanizates.

**Figure 13:**
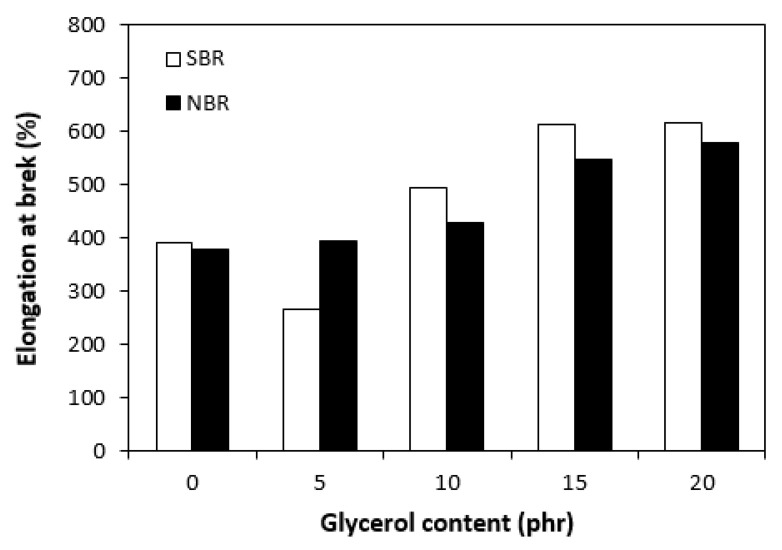
Influence of glycerol content on elongation at break of vulcanizates.

**Figure 14:**
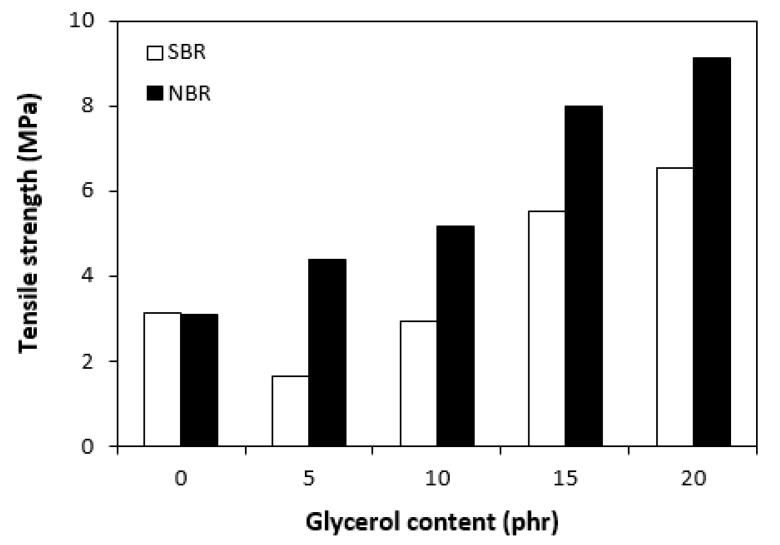
Influence of glycerol content on tensile strength of vulcanizates.

The dependences of the modulus (M300) (Figure 11) and hardness (Figure 12) of the vulcanizates on glycerol content were in close correlation with the dependences of cross-link density. The modulus (M300) of the vulcanizates based on NBR reached the maximum at 10 phr of glycerol before declining, following the trend of cross-link density (Figure 6). The lower cross-link density of the vulcanizates based on SBR was responsible for their lower modulus. As the application of glycerol in the SBR-based rubber compounds resulted in a slight decrease in the cross-linking degree, the modulus (M300) of the corresponding vulcanizates containing plasticizers was also lower than that of the reference. The lower cross-link density of the vulcanizates based on SBR was also responsible for the lower hardness of the equivalent vulcanizates (Figure 12). The influence of glycerol content on hardness was less visible, though it could be stated that the hardness of both vulcanizate types with the maximum glycerol content was lower than that of the reference. On the other hand, the application of plasticizers resulted in an increase in the elongation at break of both vulcanizate types (Figure 13). A higher elongation at break (with the exception of the sample with 5 phr of glycerol) was found in the vulcanizates based on SBR, which can be attributed to their lower cross-link density. In general, the lower the cross-link density, the higher the mobility and elasticity of rubber chain segments, and the elongation at break thus increased. However, it must be noted that the physical–mechanical properties of vulcanizates are dependent on not only the cross-link density but also the structure and nature of the rubber. For example, SBR contains 23.5% of styrene, which is a thermoplastic component of the rubber. On the other hand, NBR contains roughly 33% of acrylonitrile as its thermoplastic element. As mentioned in the introduction, the higher the amount of the thermoplastic component, the better the processing characteristics but the worse the elastic properties of rubbers. Thus, it can be concluded that the higher the elongation at break of the vulcanizates based on SBR could have been caused by the combination of a lower cross-link density and a higher amount of rubbery butadiene structural units. As seen in Figure 14, the tensile strength of the vulcanizate based on SBR with 5 phr of glycerol first decreased compared with the reference, and then a positive effect of glycerol on tensile strength was recorded. The tensile strength of the vulcanizate with a maximum glycerol content was almost twofold higher than that of the vulcanizate without plasticizers (the tensile strength increased from 3 MPa for the reference up to 6.5 MPa for the maximally filled vulcanizate). Even though the tensile strength of the reference vulcanizates based on NBR and SBR were almost the same, the tensile strength of the vulcanizates based on NBR with added glycerol was higher than that of the equivalent vulcanizates based on SBR. At the maximum glycerol content, there was a threefold increase in the tensile strength compared with the reference (from 3 MPa for the glycerol-free sample up to 9 MPa for the vulcanizate with the maximum plasticizer content). It is apparent that the application of glycerol resulted in the improvement of the tensile characteristics for both vulcanizate types. A higher enhancement of the tensile strength was recorded for the vulcanizates based on NBR. 

### 3.4. Morphology

**Figure 15:**
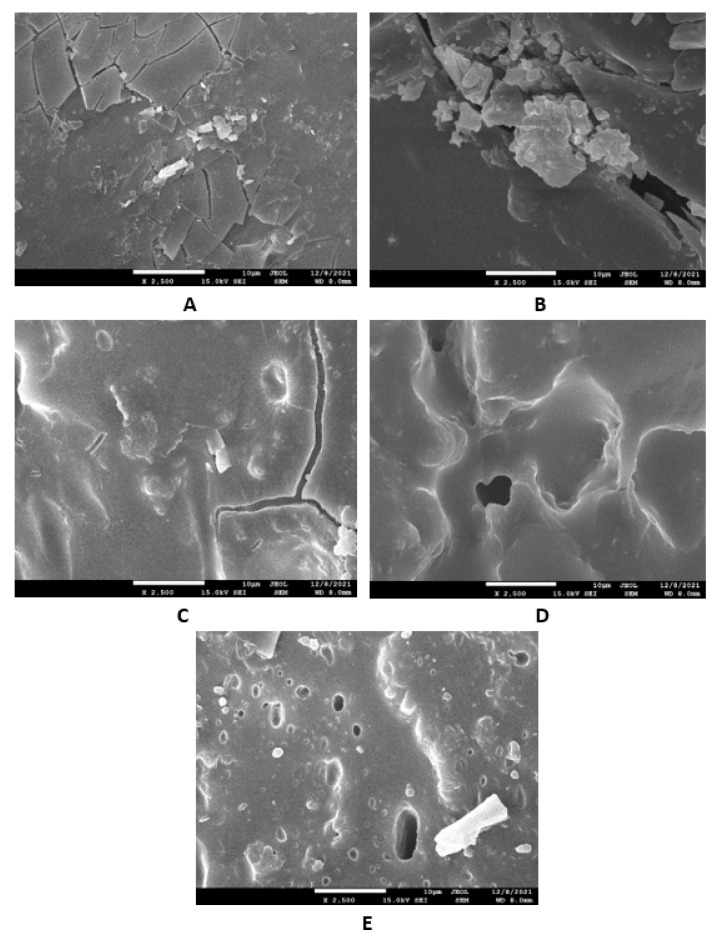
SEM images of vulcanizates based on SBR: (**A**) reference vulcanizate without glycerol, (**B**) vulcanizate with 5 phr of glycerol, (**C**) vulcanizate with 10 phr of glycerol, (**D**) vulcanizate with 15 phr of glycerol, and (**E**) vulcanizate with 20 phr of glycerol.

**Figure 16:**
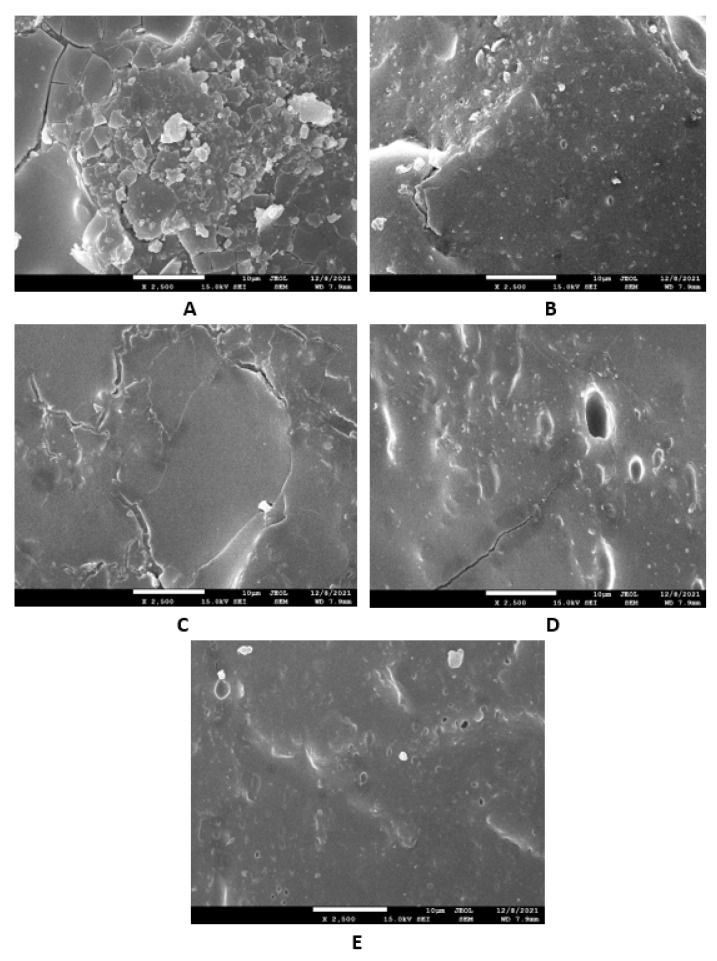
SEM images of vulcanizates based on NBR: (**A**) reference vulcanizate without glycerol, (**B**) vulcanizate with 5 phr of glycerol, (**C**) vulcanizate with 10 phr of glycerol, (**D**) vulcanizate with 15 phr of glycerol, and (**E**) vulcanizate with 20 phr of glycerol.

**Figure 17:**
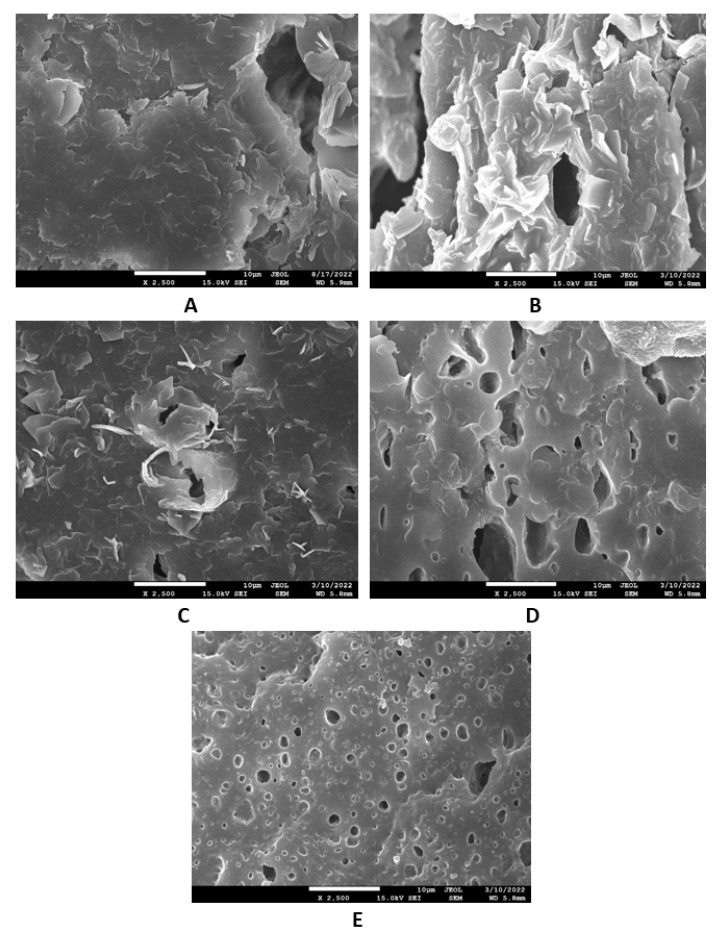
SEM images of vulcanizates based on SBR after washing in boiling water: (**A**) reference vulcanizate without glycerol, (**B**) vulcanizate with 5 phr of glycerol, (**C**) vulcanizate with 10 phr of glycerol, (**D**) vulcanizate with 15 phr of glycerol, and (**E**) vulcanizate with 20 phr of glycerol.

**Figure 18:**
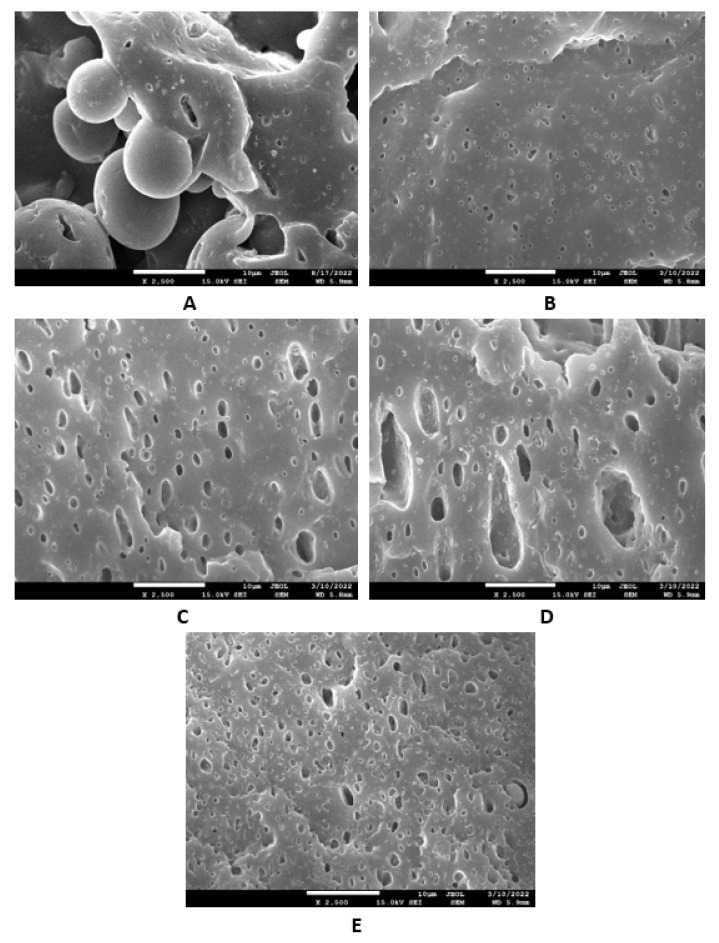
SEM images of vulcanizates based on NBR after washing in boiling water: (**A**) reference vulcanizate without glycerol, (**B**) vulcanizate with 5 phr of glycerol, (**C**) vulcanizate with 10 phr of glycerol, (**D**) vulcanizate with 15 phr of glycerol, and (**E**) vulcanizate with 20 phr of glycerol.

The study of the microstructure and morphology of the surface fractures of the vulcanizates was conducted with a scanning electron microscope. SEM images of the vulcanizates based on SBR are presented in Figure 15A–E. Figure 15A shows that lignosulfonate formed small and large agglomerates in the reference vulcanizate. The mutual adhesion between the rubber and the filler seemed to be not very good. The same statement can also be applied to the vulcanizate with 5 phr of glycerol (Figure 15B). The presence of agglomerates suggests that the dispersion of the filler and the mutual compatibility between the filler and the rubber in the filler–rubber interface was very poor. Increasing the content of glycerol led to better adhesion between the two components and the better dispersion and distribution of lignosulfonate within the rubber matrix (Figure 15C–E). Similarly, SEM images of the surface fracture of the reference NBR-based vulcanizate showed that the dispersion, distribution, and mutual compatibility between the rubber matrix and lignosulfonate were weak (Figure 16A). The addition of glycerol led to improvements of the samples’ homogeneity, lignosulfonate’s dispersion within the rubber matrix, and the mutual adhesion in the interface (Figure 16B–E). The surface structures became smoother and more compact, with no evident structural defects. To deeply investigate the dispersion and distribution of the filler within the rubber matrices, the surface morphology of the vulcanizates was also studied after the vulcanizates were washed in boiling water for 2 h. As lignosulfonate is water-soluble, it was extracted from the surface structure by boiling water. SEM images of vulcanizates after washing in boiling water are presented in Figure 17A–E (for the vulcanizates based on SBR) and Figure 18A–E (for the vulcanizates based on NBR). These figures show that the application of glycerol resulted in the much better dispersion and distribution of the lignosulfonate within the rubber matrices and the formation of smaller filler domains. However, it can be said that the vulcanizates based on SBR with 5 phr of glycerol still exhibited a non-homogeneous surface structure. Accordingly, the higher the amount of glycerol, the more homogeneous the structure. The filler domains became smaller and more uniformly distributed within the rubber matrix. Glycerol is a hydrophilic, low-molecular-weight plasticizer with polar hydroxyl groups, and it is assumed that it can reduce the softening point of a biopolymer filler, which has polar functional groups [39]. The improvements of adhesion and homogeneity between the rubber and the filler can be attributed to the softening effect of the glycerol on the lignosulfonate and the adjustment of the filler viscosity to a level closer to the viscosity of the rubber matrix during processing. The softened lignosulfonate could be then better dispersed and distributed within the rubber matrix. The amount of filler agglomerates was significantly reduced as lignosulfonate formed softer domains. Softer domains with lower rigidity more easily deform when samples are exposed to external deformation strains and can thus behave similarly to reinforcing fillers [40,41]. This can be regarded as the main point contributing to the increase in tensile strength. Comparing the SEM images of both vulcanizate types showed that the vulcanizates based on NBR demonstrated a more homogenous and compact structure, as well as a better distribution of the filler domains. This could be attributed to the presence of polar acrylonitrile structural units in NBR, owing to which NBR is a polar rubber. As lignosulfonate and glycerol are polar materials, their compatibility with polar rubber is higher than non-polar SBR, which corresponds with the higher observed tensile characteristics of the NBR-based vulcanizates with the presence of glycerol. It is also apparent that the NBR-based vulcanizate with the maximum glycerol content exhibited the most homogenous structure with the smallest filler domains (Figure 18E), so it also had the highest tensile strength. The experimentally obtained cross-link density, morphology, and rheology results also correlated very well with the physical–mechanical properties of the vulcanizates. 

### 3.5. Dynamic Mechanical Analysis

**Figure 19:**
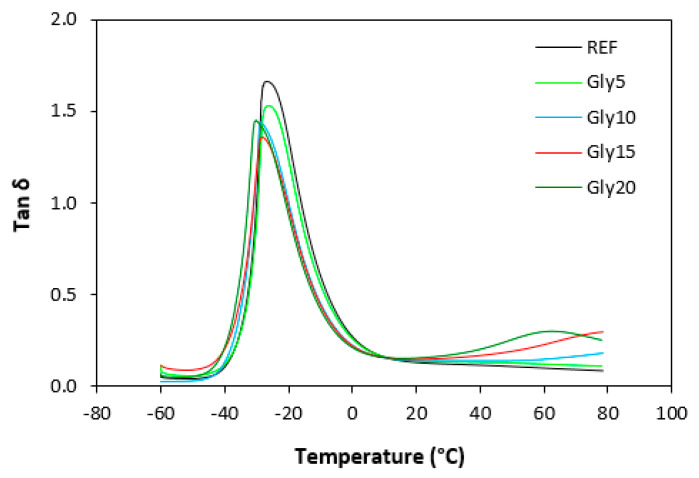
Temperature dependences of *tan δ* for vulcanizates based on SBR.

**Figure 20:**
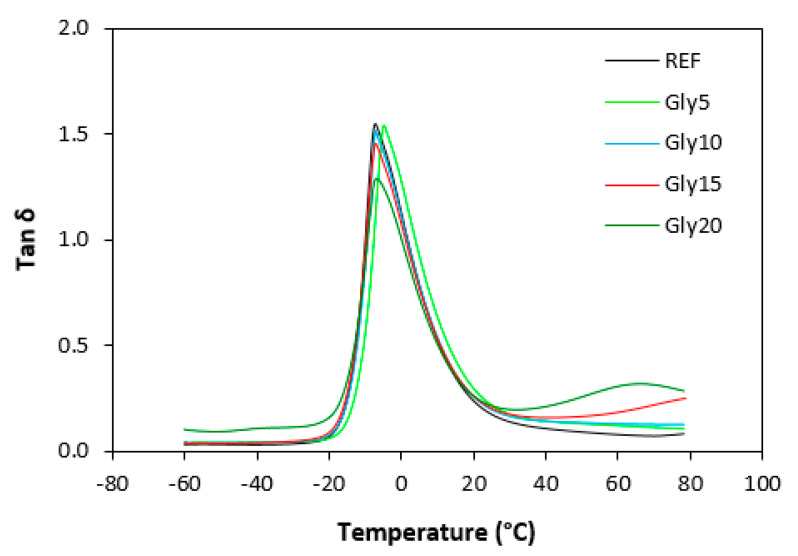
Temperature dependences of *tan δ* for vulcanizates based on NBR.

Dynamic mechanical analysis was performed to assess the influence of glycerol on the viscoelastic properties of the vulcanizates in dependence on temperature. The storage modulus (*G*′) and loss modulus (*G*″) for both types of vulcanizates at 0 °C, 20 °C and 60 °C are summarized in Table 1 and Table 2, and the glass transition temperatures and *tan δ* at the given temperatures are presented in Table 3 and Table 4. Table 1 shows that the storage modulus of the vulcanizates based on SBR slightly decreased with the increase in temperature, though little influence of the glycerol content on *G*′ was recorded. Similarly, the loss modulus showed a slight decreasing tendency with increases in temperature. The effect of glycerol content on *G*″ was more visible at high temperatures (60 °C). Compared with the reference, the loss modulus of the vulcanizate with the maximum glycerol amount increased by roughly twofold. A decreasing tendency of the storage and loss moduli with temperature increase was also recorded for the vulcanizates based on NBR (Table 2). Very significant decreases in both parameters were observed with the change in temperature from 0 to 20 °C. The storage modulus decreased by more than two times when the temperature increased from 0 °C to 20 °C. The loss modulus decreased from more than 20 MPa at 0 °C to above 2 MPa at 20 °C. The lowest *G*′ and *G*″ were found in both vulcanizate types at 60 °C. The temperature dependences of *tan δ* for the vulcanizates based on SBR are presented in Figure 19, and those for the vulcanizates based on NBR are depicted in Figure 20. The peak maximum on the curves defines the glass transition temperature, and it is shown that the *Tg* of the vulcanizates based NBR only moved in a very narrow temperature range (Table 4). The *Tg* of the vulcanizates based on SBR was lower, which indicates the better low-temperature elastic properties of those vulcanizates (Table 3). The glass transition temperature of the SBR-based vulcanizates with a higher glycerol content was slightly lower compared with the reference, which suggests that the application of glycerol could improve the dynamic characteristics of vulcanizates at low temperatures. However, it must be noted that the difference in *Tg* between the reference sample and the vulcanizate with the maximum plasticizer content was only 3 °C, which is not enough to clearly identify the influence of glycerol on the glass transition temperature. The values of *tan δ* for the vulcanizates at different temperatures are summarized in Table 3 and Table 4. It can be stated that the higher temperature, the lower the *tan δ* for both vulcanizate types with a low glycerol content (up to 10 phr). The effect of temperature was less visible for the vulcanizates with a higher glycerol content. The vulcanizates based on both SBR and NBR with high glycerol contents (15 and 20 phr) exhibited higher *tan δ* values at 60 °C. However, in general, it can be stated that no significant influence of glycerol on the dynamic mechanical properties of the vulcanizates was recorded. The *Tg* of the vulcanizates based on SBR was lower than that of the equivalent vulcanizates based on NBR, which can be attributed to the microstructure of both elastomers and the lower cross-link density of the vulcanizates based on SBR. As already mentioned, SBR contains a smaller amount of thermoplastic components than NBR, so it also has a higher amount of rubbery butadiene units, which are responsible for the elastic properties of vulcanizates. Additionally, the lower cross-link density led to the higher elasticity and mobility of the rubber chains and thus contributed to lower *Tg*.

## 4. Conclusions

Calcium lignosulfonate was incorporated into rubber compounds based on SBR and NBR. Glycerol—a cheap, non-toxic, and environmentally friendly plasticizer—was added to the rubber formulations in a concentration scale ranging from 5 to 20 phr. A sulfur-based curing system was used for the cross-linking of the rubber formulations.

The results revealed that the presence of glycerol influenced the shape and inclination of the curing isotherms, which was subsequently reflected in changes in curing characteristics. The maximum torque, minimum torque, and torque difference were found to decrease with increases in glycerol content, which clearly indicate its strong plasticizing effect on the rubber compounds. Rheological measurements confirmed the presumption that the higher the amount of glycerol, the lower the viscosity of the rubber compounds. Morphological analysis revealed that the addition of glycerol resulted in the better dispersion and distribution of the biopolymer filler within the rubber matrices and also contributed to the improvements of adhesion and compatibility between the rubber and the filler in the filler–rubber interface. Higher levels of homogeneity and compatibility between the rubber and lignosulfonate were observed in the surface structure of the vulcanizates based on NBR, very likely due to the compatibility of the polarity of the rubber, the filler, and the plasticizer. This was subsequently reflected in the larger improvement of the tensile strength of the vulcanizates based on NBR compared with those based on SBR. The results of the experiments demonstrated a very good correlation among the rheological and morphological measurements, cross-link density, and physical–mechanical properties of the vulcanizates. On the other hand, almost no changes in the dynamic mechanical characteristics of the vulcanizates were recorded regarding the dependence on plasticizer content. 

## Figures and Tables

**Table 1:** Storage modulus (*G*′) and loss modulus (*G*″) for vulcanizates based on SBR at different temperatures.

Glycerol (phr)	*G*′ (MPa) (0 °C)	*G*′ (MPa) (20 °C)	*G*′ (MPa) (60 °C)	*G*″ (MPa) (0 °C)	*G*″ (MPa) (20 °C)	*G*″ (MPa) (60 °C)
0	6.17	5.06	4.25	1.65	0.64	0.41
5	6.78	5.66	4.75	1.80	0.81	0.60
10	7.82	6.47	5.16	1.77	0.93	0.78
15	9.02	7.15	4.35	2.01	1.07	1.01
20	7.94	6.27	2.93	1.67	0.95	0.87

**Table 2:** Storage modulus (*G*′) and loss modulus (*G*″) for vulcanizates based on NBR at different temperatures.

Glycerol (phr)	*G*′ (MPa) (0 °C)	*G*′ (MPa) (20 °C)	*G*′ (MPa) (60 °C)	*G*″ (MPa) (0 °C)	*G*″ (MPa) (20 °C)	*G*″ (MPa) (60 °C)
0	21.79	8.20	6.43	24.74	1.97	0.51
5	28.19	9.46	6.81	35.96	2.79	0.81
10	26.70	10.12	7.08	29.83	2.64	0.92
15	25.84	9.78	6.05	27.70	2.51	1.11
20	24.22	8.97	3.84	24.55	2.39	1.19

**Table 3:** Glass transition temperature and *tan δ* for vulcanizates based on SBR.

Glycerol (phr)	*Tg* (°C)	*tan δ* (0 °C)	*tan δ* (20 °C)	*tan δ* (60 °C)
0	−26.8	0.27	0.13	0.10
5	−26.1	0.27	0.14	0.13
10	−28.6	0.23	0.14	0.15
15	−28.2	0.22	0.15	0.23
20	−30.2	0.21	0.15	0.30

**Table 4:** Glass transition temperature and *tan δ* for vulcanizates based on NBR.

Glycerol (phr)	*Tg* (°C)	*tan δ* (0 °C)	*tan δ* (20 °C)	*tan δ* (60 °C)
0	−7.4	1.14	0.24	0.08
5	−5.1	1.28	0.30	0.12
10	−7	1.12	0.26	0.13
15	−7.4	1.07	0.26	0.18
20	−6.8	1.01	0.27	0.31

## Data Availability

Not applicable.
